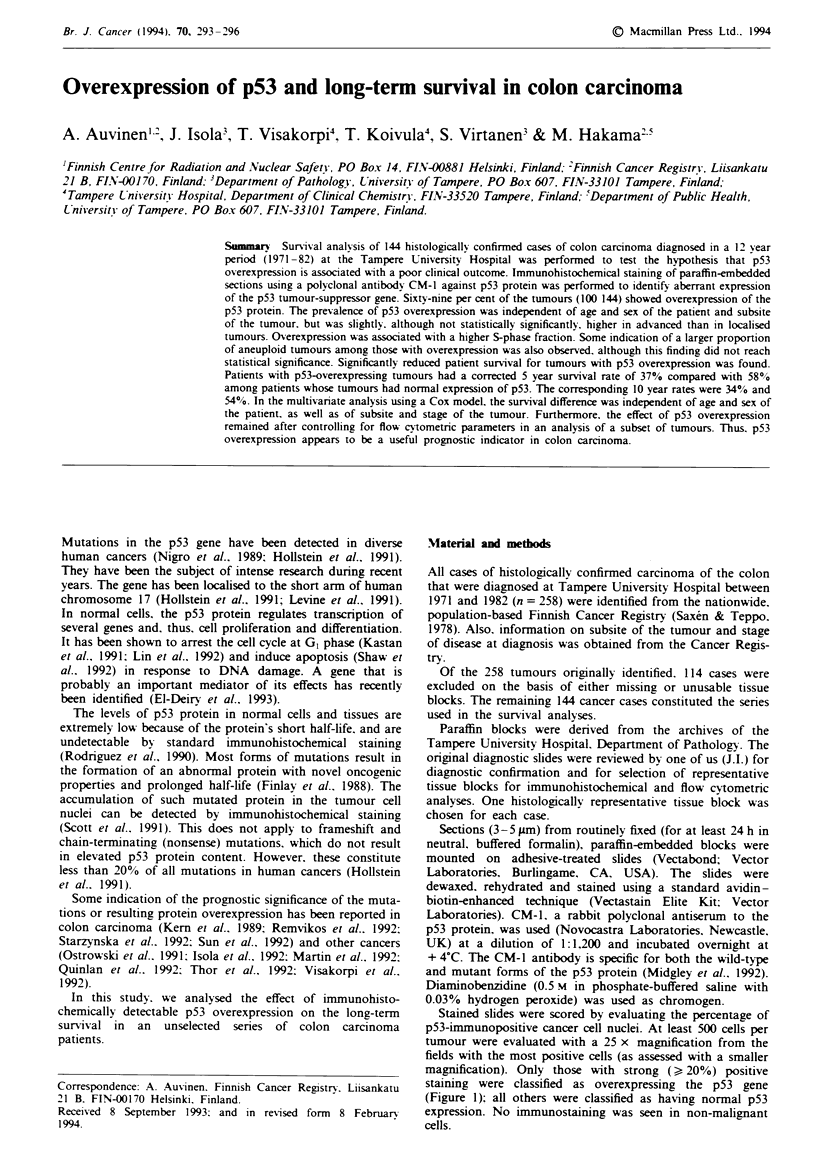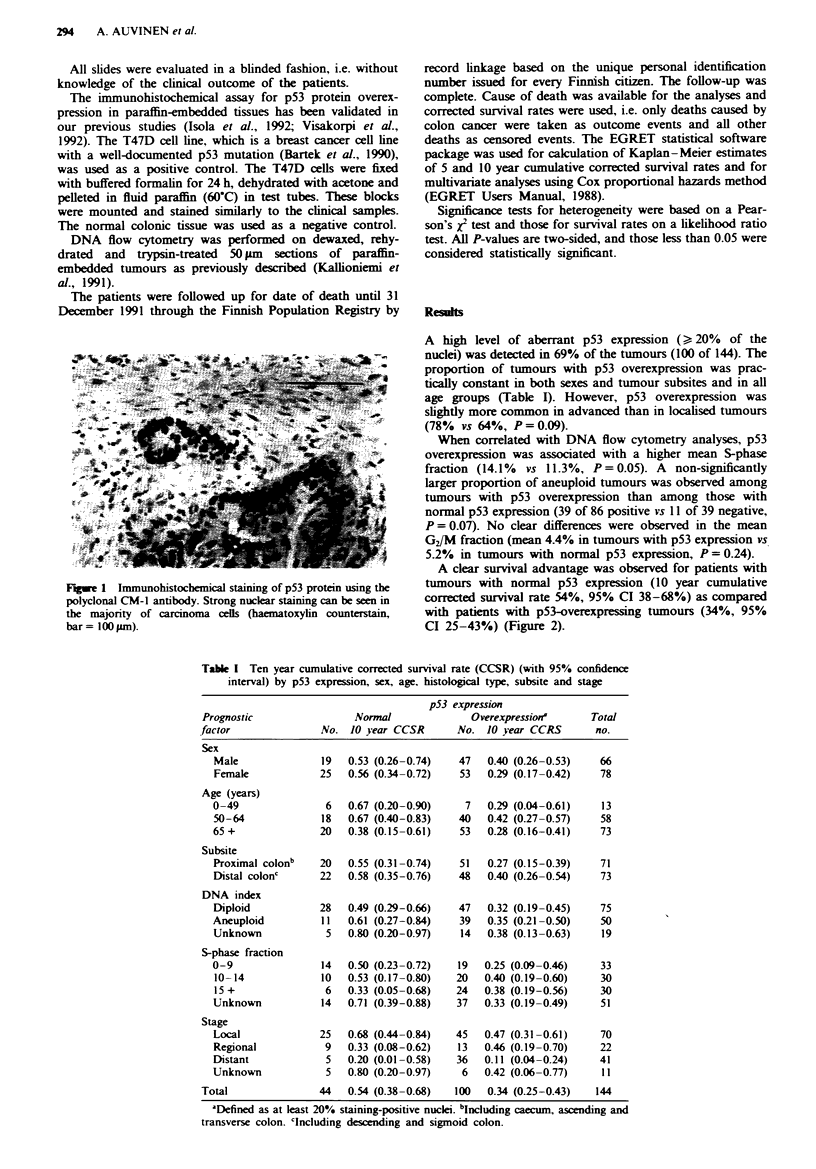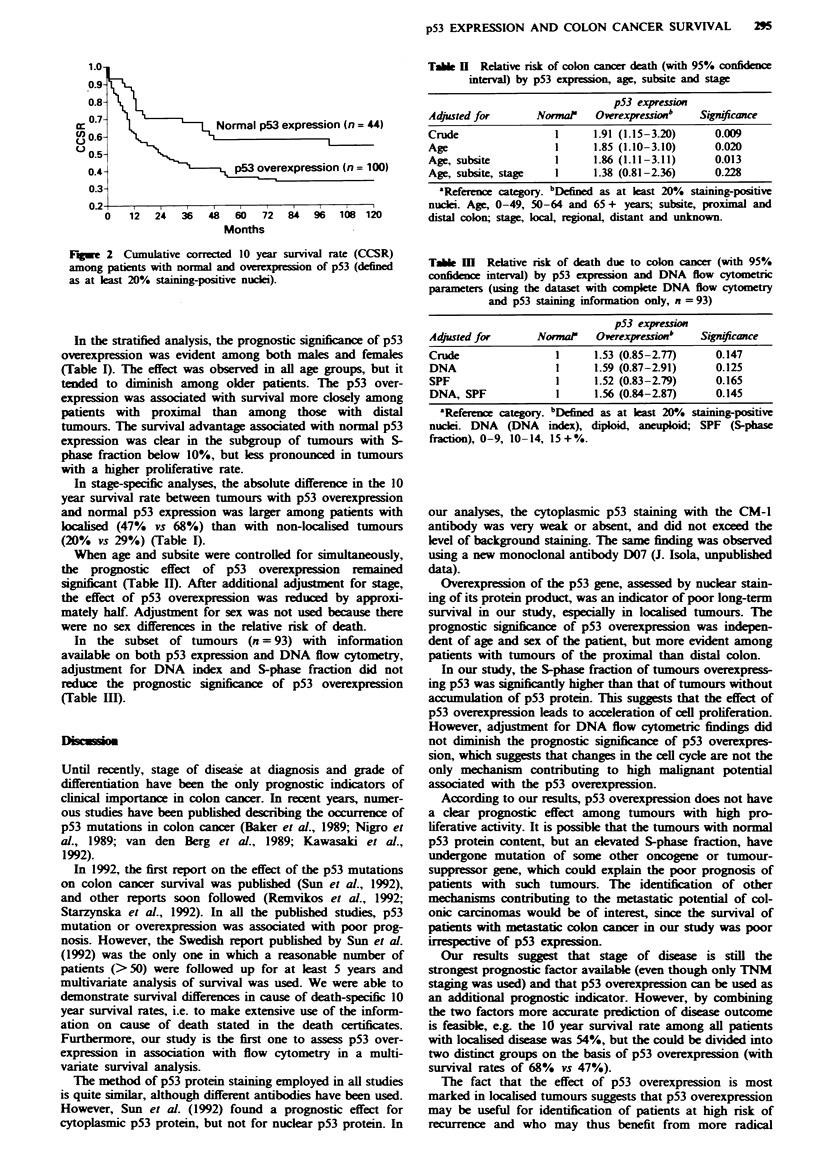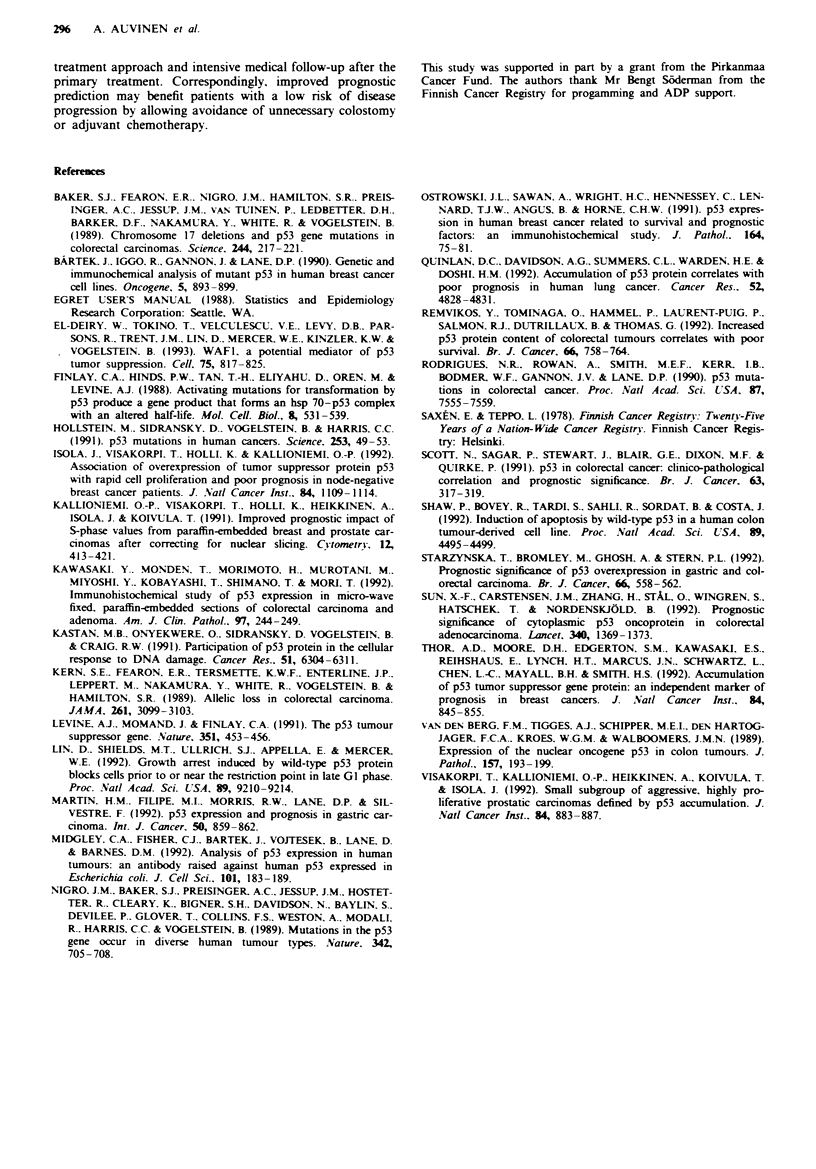# Overexpression of p53 and long-term survival in colon carcinoma.

**DOI:** 10.1038/bjc.1994.295

**Published:** 1994-08

**Authors:** A. Auvinen, J. Isola, T. Visakorpi, T. Koivula, S. Virtanen, M. Hakama

**Affiliations:** Finnish Centre for Radiation and Nuclear Safety, Helsinki, Finland.

## Abstract

**Images:**


Br. J. Cancer (1994), 70, 293-296 © Macmillan Press Ltd., 1994

### Overexpression of p53 and long-term survival in colon carcinoma

A. Auvinen[1,2], J. Isola[3], T. Visakorpi[4], T. Koivula[4], S. Virtanen[3] & M. Hakama[2,5]

[1]Finnish Centre for Radiation and Nuclear Safety, PO Box 14, FIN-00881 Helsinki, Finland; [2]Finnish Cancer Registry, Liisankatu 21 B, FIN-00170, Finland; [3]Department of Pathology, University of Tampere, PO Box 607, FIN-33101 Tampere, Finland; [4]Tampere University Hospital, Department of Clinical Chemistry, FIN-33520 Tampere, Finland; [5]Department of Public Health, University of Tampere, PO Box 607, FIN-33101 Tampere, Finland.

**Summary** Survival analysis of 144 histologically confirmed cases of colon carcinoma diagnosed in a 12 year period (1971-82) at the Tampere University Hospital was performed to test the hypothesis that p53 overexpression is associated with a poor clinical outcome. Immunohistochemical staining of paraffin-embedded sections using a polyclonal antibody CM-1 against p53 protein was performed to identify aberrant expression of the p53 tumour-suppressor gene. Sixty-nine per cent of the tumours (100 144) showed overexpression of the p53 protein. The prevalence of p53 overexpression was independent of age and sex of the patient and subsite of the tumour, but was slightly, although not statistically significantly, higher in advanced than in localised tumours. Overexpression was associated with a higher S-phase fraction. Some indication of a larger proportion of aneuploid tumours among those with overexpression was also observed, although this finding did not reach statistical significance. Significantly reduced patient survival for tumours with p53 overexpression was found. Patients with p53-overexpressing tumours had a corrected 5 year survival rate of 37% compared with 58% among patients whose tumours had normal expression of p53. The corresponding 10 year rates were 34% and 54%. In the multivariate analysis using a Cox model, the survival difference was independent of age and sex of the patient, as well as of subsite and stage of the tumour. Furthermore, the effect of p53 overexpression remained after controlling for flow cytometric parameters in an analysis of a subset of tumours. Thus, p53 overexpression appears to be a useful prognostic indicator in colon carcinoma.

Mutations in the p53 gene have been detected in diverse human cancers (Nigro *et al.*, 1989; Hollstein *et al.*, 1991). They have been the subject of intense research during recent years. The gene has been localised to the short arm of human chromosome 17 (Hollstein *et al.*, 1991; Levine *et al.*, 1991). In normal cells, the p53 protein regulates transcription of several genes and, thus, cell proliferation and differentiation. It has been shown to arrest the cell cycle at $G_1$ phase (Kastan *et al.*, 1991; Lin *et al.*, 1992) and induce apoptosis (Shaw *et al.*, 1992) in response to DNA damage. A gene that is probably an important mediator of its effects has recently been identified (El-Deiry *et al.*, 1993).

The levels of p53 protein in normal cells and tissues are extremely low because of the protein's short half-life, and are undetectable by standard immunohistochemical staining (Rodriguez *et al.*, 1990). Most forms of mutations result in the formation of an abnormal protein with novel oncogenic properties and prolonged half-life (Finlay *et al.*, 1988). The accumulation of such mutated protein in the tumour cell nuclei can be detected by immunohistochemical staining (Scott *et al.*, 1991). This does not apply to frameshift and chain-terminating (nonsense) mutations, which do not result in elevated p53 protein content. However, these constitute less than 20% of all mutations in human cancers (Hollstein *et al.*, 1991).

Some indication of the prognostic significance of the mutations or resulting protein overexpression has been reported in colon carcinoma (Kern *et al.*, 1989; Remvikos *et al.*, 1992; Starzynska *et al.*, 1992; Sun *et al.*, 1992) and other cancers (Ostrowski *et al.*, 1991; Isola *et al.*, 1992; Martin *et al.*, 1992; Quinlan *et al.*, 1992; Thor *et al.*, 1992; Visakorpi *et al.*, 1992).

In this study, we analysed the effect of immunohistochemically detectable p53 overexpression on the long-term survival in an unselected series of colon carcinoma patients.

Correspondence: A. Auvinen, Finnish Cancer Registry, Liisankatu 21 B, FIN-00170 Helsinki, Finland.
Received 8 September 1993; and in revised form 8 February 1994.

### Material and methods

All cases of histologically confirmed carcinoma of the colon that were diagnosed at Tampere University Hospital between 1971 and 1982 ($n = 258$) were identified from the nationwide, population-based Finnish Cancer Registry (Saxén & Teppo, 1978). Also, information on subsite of the tumour and stage of disease at diagnosis was obtained from the Cancer Registry.

Of the 258 tumours originally identified, 114 cases were excluded on the basis of either missing or unusable tissue blocks. The remaining 144 cancer cases constituted the series used in the survival analyses.

Paraffin blocks were derived from the archives of the Tampere University Hospital, Department of Pathology. The original diagnostic slides were reviewed by one of us (J.I.) for diagnostic confirmation and for selection of representative tissue blocks for immunohistochemical and flow cytometric analyses. One histologically representative tissue block was chosen for each case.

Sections (3-5 μm) from routinely fixed (for at least 24 h in neutral, buffered formalin), paraffin-embedded blocks were mounted on adhesive-treated slides (Vectabond; Vector Laboratories, Burlingame, CA, USA). The slides were dewaxed, rehydrated and stained using a standard avidin-biotin-enhanced technique (Vectastain Elite Kit; Vector Laboratories). CM-1, a rabbit polyclonal antiserum to the p53 protein, was used (Novocastra Laboratories, Newcastle, UK) at a dilution of 1:1,200 and incubated overnight at + 4°C. The CM-1 antibody is specific for both the wild-type and mutant forms of the p53 protein (Midgley *et al.*, 1992). Diaminobenzidine (0.5 M in phosphate-buffered saline with 0.03% hydrogen peroxide) was used as chromogen.

Stained slides were scored by evaluating the percentage of p53-immunopositive cancer cell nuclei. At least 500 cells per tumour were evaluated with a 25 × magnification from the fields with the most positive cells (as assessed with a smaller magnification). Only those with strong (≥ 20%) positive staining were classified as overexpressing the p53 gene (Figure 1); all others were classified as having normal p53 expression. No immunostaining was seen in non-malignant cells.

All slides were evaluated in a blinded fashion, i.e. without knowledge of the clinical outcome of the patients.

The immunohistochemical assay for p53 protein overexpression in paraffin-embedded tissues has been validated in our previous studies (Isola et al., 1992; Visakorpi et al., 1992). The T47D cell line, which is a breast cancer cell line with a well-documented p53 mutation (Bartek et al., 1990), was used as a positive control. The T47D cells were fixed with buffered formalin for 24 h, dehydrated with acetone and pelleted in fluid paraffin (60°C) in test tubes. These blocks were mounted and stained similarly to the clinical samples. The normal colonic tissue was used as a negative control.

DNA flow cytometry was performed on dewaxed, rehydrated and trypsin-treated 50 µm sections of paraffin-embedded tumours as previously described (Kallioniemi et al., 1991).

The patients were followed up for date of death until 31 December 1991 through the Finnish Population Registry by record linkage based on the unique personal identification number issued for every Finnish citizen. The follow-up was complete. Cause of death was available for the analyses and corrected survival rates were used, i.e. only deaths caused by colon cancer were taken as outcome events and all other deaths as censored events. The EGRET statistical software package was used for calculation of Kaplan-Meier estimates of 5 and 10 year cumulative corrected survival rates and for multivariate analyses using Cox proportional hazards method (EGRET Users Manual, 1988).

Significance tests for heterogeneity were based on a Pearson's $\chi^2$ test and those for survival rates on a likelihood ratio test. All $P$-values are two-sided, and those less than 0.05 were considered statistically significant.

**Figure 1** Immunohistochemical staining of p53 protein using the polyclonal CM-1 antibody. Strong nuclear staining can be seen in the majority of carcinoma cells (haematoxylin counterstain, bar = 100 µm).

### Results

A high level of aberrant p53 expression (⩾ 20% of the nuclei) was detected in 69% of the tumours (100 of 144). The proportion of tumours with p53 overexpression was practically constant in both sexes and tumour subsites and in all age groups (Table I). However, p53 overexpression was slightly more common in advanced than in localised tumours (78% vs 64%, $P = 0.09$).

When correlated with DNA flow cytometry analyses, p53 overexpression was associated with a higher mean S-phase fraction (14.1% vs 11.3%, $P = 0.05$). A non-significantly larger proportion of aneuploid tumours was observed among tumours with p53 overexpression than among those with normal p53 expression (39 of 86 positive vs 11 of 39 negative, $P = 0.07$). No clear differences were observed in the mean $G_2$/M fraction (mean 4.4% in tumours with p53 expression vs 5.2% in tumours with normal p53 expression, $P = 0.24$).

A clear survival advantage was observed for patients with tumours with normal p53 expression (10 year cumulative corrected survival rate 54%, 95% CI 38-68%) as compared with patients with p53-overexpressing tumours (34%, 95% CI 25-43%) (Figure 2).

**Table I** Ten year cumulative corrected survival rate (CCSR) (with 95% confidence interval) by p53 expression, sex, age, histological type, subsite and stage

| | p53 expression | | | | |
|---|---|---|---|---|---|
| | Normal | | Overexpression[a] | | |
| Prognostic factor | No. | 10 year CCSR | No. | 10 year CCRS | Total no. |
| Sex | | | | | |
| Male | 19 | 0.53 (0.26-0.74) | 47 | 0.40 (0.26-0.53) | 66 |
| Female | 25 | 0.56 (0.34-0.72) | 53 | 0.29 (0.17-0.42) | 78 |
| Age (years) | | | | | |
| 0-49 | 6 | 0.67 (0.20-0.90) | 7 | 0.29 (0.04-0.61) | 13 |
| 50-64 | 18 | 0.67 (0.40-0.83) | 40 | 0.42 (0.27-0.57) | 58 |
| 65 + | 20 | 0.38 (0.15-0.61) | 53 | 0.28 (0.16-0.41) | 73 |
| Subsite | | | | | |
| Proximal colon[b] | 20 | 0.55 (0.31-0.74) | 51 | 0.27 (0.15-0.39) | 71 |
| Distal colon[c] | 22 | 0.58 (0.35-0.76) | 48 | 0.40 (0.26-0.54) | 73 |
| DNA index | | | | | |
| Diploid | 28 | 0.49 (0.29-0.66) | 47 | 0.32 (0.19-0.45) | 75 |
| Aneuploid | 11 | 0.61 (0.27-0.84) | 39 | 0.35 (0.21-0.50) | 50 |
| Unknown | 5 | 0.80 (0.20-0.97) | 14 | 0.38 (0.13-0.63) | 19 |
| S-phase fraction | | | | | |
| 0-9 | 14 | 0.50 (0.23-0.72) | 19 | 0.25 (0.09-0.46) | 33 |
| 10-14 | 10 | 0.53 (0.17-0.80) | 20 | 0.40 (0.19-0.60) | 30 |
| 15 + | 6 | 0.33 (0.05-0.68) | 24 | 0.38 (0.19-0.56) | 30 |
| Unknown | 14 | 0.71 (0.39-0.88) | 37 | 0.33 (0.19-0.49) | 51 |
| Stage | | | | | |
| Local | 25 | 0.68 (0.44-0.84) | 45 | 0.47 (0.31-0.61) | 70 |
| Regional | 9 | 0.33 (0.08-0.62) | 13 | 0.46 (0.19-0.70) | 22 |
| Distant | 5 | 0.20 (0.01-0.58) | 36 | 0.11 (0.04-0.24) | 41 |
| Unknown | 5 | 0.80 (0.20-0.97) | 6 | 0.42 (0.06-0.77) | 11 |
| Total | 44 | 0.54 (0.38-0.68) | 100 | 0.34 (0.25-0.43) | 144 |

[a]Defined as at least 20% staining-positive nuclei. [b]Including caecum, ascending and transverse colon. [c]Including descending and sigmoid colon.

**Figure 2** Cumulative corrected 10 year survival rate (CCSR) among patients with normal and overexpression of p53 (defined as at least 20% staining-positive nuclei).

In the stratified analysis, the prognostic significance of p53 overexpression was evident among both males and females (Table I). The effect was observed in all age groups, but it tended to diminish among older patients. The p53 overexpression was associated with survival more closely among patients with proximal than among those with distal tumours. The survival advantage associated with normal p53 expression was clear in the subgroup of tumours with S-phase fraction below 10%, but less pronounced in tumours with a higher proliferative rate.

In stage-specific analyses, the absolute difference in the 10 year survival rate between tumours with p53 overexpression and normal p53 expression was larger among patients with localised (47% *vs* 68%) than with non-localised tumours (20% *vs* 29%) (Table I).

When age and subsite were controlled for simultaneously, the prognostic effect of p53 overexpression remained significant (Table II). After additional adjustment for stage, the effect of p53 overexpression was reduced by approximately half. Adjustment for sex was not used because there were no sex differences in the relative risk of death.

In the subset of tumours ($n = 93$) with information available on both p53 expression and DNA flow cytometry, adjustment for DNA index and S-phase fraction did not reduce the prognostic significance of p53 overexpression (Table III).

**Table II** Relative risk of colon cancer death (with 95% confidence interval) by p53 expression, age, subsite and stage

| *Adjusted for* | *p53 expression* Normal[a] | Overexpression[b] | *Significance* |
|---|---|---|---|
| Crude | 1 | 1.91 (1.15–3.20) | 0.009 |
| Age | 1 | 1.85 (1.10–3.10) | 0.020 |
| Age, subsite | 1 | 1.86 (1.11–3.11) | 0.013 |
| Age, subsite, stage | 1 | 1.38 (0.81–2.36) | 0.228 |

[a]Reference category. [b]Defined as at least 20% staining-positive nuclei. Age, 0–49, 50–64 and 65 + years; subsite, proximal and distal colon; stage, local, regional, distant and unknown.

**Table III** Relative risk of death due to colon cancer (with 95% confidence interval) by p53 expression and DNA flow cytometric parameters (using the dataset with complete DNA flow cytometry and p53 staining information only, $n = 93$)

| *Adjusted for* | *p53 expression* Normal[a] | Overexpression[b] | *Significance* |
|---|---|---|---|
| Crude | 1 | 1.53 (0.85–2.77) | 0.147 |
| DNA | 1 | 1.59 (0.87–2.91) | 0.125 |
| SPF | 1 | 1.52 (0.83–2.79) | 0.165 |
| DNA, SPF | 1 | 1.56 (0.84–2.87) | 0.145 |

[a]Reference category. [b]Defined as at least 20% staining-positive nuclei. DNA (DNA index), diploid, aneuploid; SPF (S-phase fraction), 0–9, 10–14, 15 + %.

### Discussion

Until recently, stage of disease at diagnosis and grade of differentiation have been the only prognostic indicators of clinical importance in colon cancer. In recent years, numerous studies have been published describing the occurrence of p53 mutations in colon cancer (Baker *et al.*, 1989; Nigro *et al.*, 1989; van den Berg *et al.*, 1989; Kawasaki *et al.*, 1992).

In 1992, the first report on the effect of the p53 mutations on colon cancer survival was published (Sun *et al.*, 1992), and other reports soon followed (Remvikos *et al.*, 1992; Starzynska *et al.*, 1992). In all the published studies, p53 mutation or overexpression was associated with poor prognosis. However, the Swedish report published by Sun *et al.* (1992) was the only one in which a reasonable number of patients (>50) were followed up for at least 5 years and multivariate analysis of survival was used. We were able to demonstrate survival differences in cause of death-specific 10 year survival rates, i.e. to make extensive use of the information on cause of death stated in the death certificates. Furthermore, our study is the first one to assess p53 overexpression in association with flow cytometry in a multivariate survival analysis.

The method of p53 protein staining employed in all studies is quite similar, although different antibodies have been used. However, Sun *et al.* (1992) found a prognostic effect for cytoplasmic p53 protein, but not for nuclear p53 protein. In our analyses, the cytoplasmic p53 staining with the CM-1 antibody was very weak or absent, and did not exceed the level of background staining. The same finding was observed using a new monoclonal antibody D07 (J. Isola, unpublished data).

Overexpression of the p53 gene, assessed by nuclear staining of its protein product, was an indicator of poor long-term survival in our study, especially in localised tumours. The prognostic significance of p53 overexpression was independent of age and sex of the patient, but more evident among patients with tumours of the proximal than distal colon.

In our study, the S-phase fraction of tumours overexpressing p53 was significantly higher than that of tumours without accumulation of p53 protein. This suggests that the effect of p53 overexpression leads to acceleration of cell proliferation. However, adjustment for DNA flow cytometric findings did not diminish the prognostic significance of p53 overexpression, which suggests that changes in the cell cycle are not the only mechanism contributing to high malignant potential associated with the p53 overexpression.

According to our results, p53 overexpression does not have a clear prognostic effect among tumours with high proliferative activity. It is possible that the tumours with normal p53 protein content, but an elevated S-phase fraction, have undergone mutation of some other oncogene or tumour-suppressor gene, which could explain the poor prognosis of patients with such tumours. The identification of other mechanisms contributing to the metastatic potential of colonic carcinomas would be of interest, since the survival of patients with metastatic colon cancer in our study was poor irrespective of p53 expression.

Our results suggest that stage of disease is still the strongest prognostic factor available (even though only TNM staging was used) and that p53 overexpression can be used as an additional prognostic indicator. However, by combining the two factors more accurate prediction of disease outcome is feasible, e.g. the 10 year survival rate among all patients with localised disease was 54%, but the could be divided into two distinct groups on the basis of p53 overexpression (with survival rates of 68% *vs* 47%).

The fact that the effect of p53 overexpression is most marked in localised tumours suggests that p53 overexpression may be useful for identification of patients at high risk of recurrence and who may thus benefit from more radical treatment approach and intensive medical follow-up after the primary treatment. Correspondingly, improved prognostic prediction may benefit patients with a low risk of disease progression by allowing avoidance of unnecessary colostomy or adjuvant chemotherapy.

This study was supported in part by a grant from the Pirkanmaa Cancer Fund. The authors thank Mr Bengt Söderman from the Finnish Cancer Registry for progamming and ADP support.

### References

BAKER, S.J., FEARON, E.R., NIGRO, J.M., HAMILTON, S.R., PREISINGER, A.C., JESSUP, J.M., VAN TUINEN, P., LEDBETTER, D.H., BARKER, D.F., NAKAMURA, Y., WHITE, R. & VOGELSTEIN, B. (1989). Chromosome 17 deletions and p53 gene mutations in colorectal carcinomas. *Science*, **244**, 217-221.

BÁRTEK, J., IGGO, R., GANNON, J. & LANE, D.P. (1990). Genetic and immunochemical analysis of mutant p53 in human breast cancer cell lines. *Oncogene*, **5**, 893-899.

EGRET USER'S MANUAL (1988). Statistics and Epidemiology Research Corporation: Seattle, WA.

EL-DEIRY, W., TOKINO, T., VELCULESCU, V.E., LEVY, D.B., PARSONS, R., TRENT, J.M., LIN, D., MERCER, W.E., KINZLER, K.W. & VOGELSTEIN, B. (1993). WAF1, a potential mediator of p53 tumor suppression. *Cell*, **75**, 817-825.

FINLAY, C.A., HINDS, P.W., TAN, T.-H., ELIYAHU, D., OREN, M. & LEVINE, A.J. (1988). Activating mutations for transformation by p53 produce a gene product that forms an hsp 70-p53 complex with an altered half-life. *Mol. Cell. Biol.*, **8**, 531-539.

HOLLSTEIN, M., SIDRANSKY, D., VOGELSTEIN, B. & HARRIS, C.C. (1991). p53 mutations in human cancers. *Science*, **253**, 49-53.

ISOLA, J., VISAKORPI, T., HOLLI, K. & KALLIONIEMI, O.-P. (1992). Association of overexpression of tumor suppressor protein p53 with rapid cell proliferation and poor prognosis in node-negative breast cancer patients. *J. Natl Cancer Inst.*, **84**, 1109-1114.

KALLIONIEMI, O.-P., VISAKORPI, T., HOLLI, K., HEIKKINEN, A., ISOLA, J. & KOIVULA, T. (1991). Improved prognostic impact of S-phase values from paraffin-embedded breast and prostate carcinomas after correcting for nuclear slicing. *Cytometry*, **12**, 413-421.

KAWASAKI, Y., MONDEN, T., MORIMOTO, H., MUROTANI, M., MIYOSHI, Y., KOBAYASHI, T., SHIMANO, T. & MORI, T. (1992). Immunohistochemical study of p53 expression in micro-wave fixed, paraffin-embedded sections of colorectal carcinoma and adenoma. *Am. J. Clin. Pathol.*, **97**, 244-249.

KASTAN, M.B., ONYEKWERE, O., SIDRANSKY, D. VOGELSTEIN, B. & CRAIG, R.W. (1991). Participation of p53 protein in the cellular response to DNA damage. *Cancer Res.*, **51**, 6304-6311.

KERN, S.E., FEARON, E.R., TERSMETTE, K.W.F., ENTERLINE, J.P., LEPPERT, M., NAKAMURA, Y., WHITE, R., VOGELSTEIN, B. & HAMILTON, S.R. (1989). Allelic loss in colorectal carcinoma. *JAMA*, **261**, 3099-3103.

LEVINE, A.J., MOMAND, J. & FINLAY, C.A. (1991). The p53 tumour suppressor gene. *Nature*, **351**, 453-456.

LIN, D., SHIELDS, M.T., ULLRICH, S.J., APPELLA, E. & MERCER, W.E. (1992). Growth arrest induced by wild-type p53 protein blocks cells prior to or near the restriction point in late G1 phase. *Proc. Natl Acad. Sci. USA*, **89**, 9210-9214.

MARTIN, H.M., FILIPE, M.I., MORRIS, R.W., LANE, D.P. & SILVESTRE, F. (1992). p53 expression and prognosis in gastric carcinoma. *Int. J. Cancer*, **50**, 859-862.

MIDGLEY, C.A., FISHER, C.J., BARTEK, J., VOJTESEK, B., LANE, D. & BARNES, D.M. (1992). Analysis of p53 expression in human tumours: an antibody raised against human p53 expressed in *Escherichia coli*. *J. Cell Sci.*, **101**, 183-189.

NIGRO, J.M., BAKER, S.J., PREISINGER, A.C., JESSUP, J.M., HOSTETTER, R., CLEARY, K., BIGNER, S.H., DAVIDSON, N., BAYLIN, S., DEVILEE, P., GLOVER, T., COLLINS, F.S., WESTON, A., MODALI, R., HARRIS, C.C. & VOGELSTEIN, B. (1989). Mutations in the p53 gene occur in diverse human tumour types. *Nature*, **342**, 705-708.

OSTROWSKI, J.L., SAWAN, A., WRIGHT, H.C., HENNESSEY, C., LENNARD, T.J.W., ANGUS, B. & HORNE, C.H.W. (1991). p53 expression in human breast cancer related to survival and prognostic factors: an immunohistochemical study. *J. Pathol.*, **164**, 75-81.

QUINLAN, D.C., DAVIDSON, A.G., SUMMERS, C.L., WARDEN, H.E. & DOSHI, H.M. (1992). Accumulation of p53 protein correlates with poor prognosis in human lung cancer. *Cancer Res.*, **52**, 4828-4831.

REMVIKOS, Y., TOMINAGA, O., HAMMEL, P., LAURENT-PUIG, P., SALMON, R.J., DUTRILLAUX, B. & THOMAS, G. (1992). Increased p53 protein content of colorectal tumours correlates with poor survival. *Br. J. Cancer*, **66**, 758-764.

RODRIGUES, N.R., ROWAN, A., SMITH, M.E.F., KERR, I.B., BODMER, W.F., GANNON, J.V. & LANE, D.P. (1990). p53 mutations in colorectal cancer. *Proc. Natl Acad. Sci. USA*, **87**, 7555-7559.

SAXÉN, E. & TEPPO, L. (1978). *Finnish Cancer Registry: Twenty-Five Years of a Nation-Wide Cancer Registry*. Finnish Cancer Registry: Helsinki.

SCOTT, N., SAGAR, P., STEWART, J., BLAIR, G.E., DIXON, M.F. & QUIRKE, P. (1991). p53 in colorectal cancer: clinico-pathological correlation and prognostic significance. *Br. J. Cancer*, **63**, 317-319.

SHAW, P., BOVEY, R., TARDI, S., SAHLI, R., SORDAT, B. & COSTA, J. (1992). Induction of apoptosis by wild-type p53 in a human colon tumour-derived cell line. *Proc. Natl Acad. Sci. USA*, **89**, 4495-4499.

STARZYNSKA, T., BROMLEY, M., GHOSH, A. & STERN, P.L. (1992). Prognostic significance of p53 overexpression in gastric and colorectal carcinoma. *Br. J. Cancer*, **66**, 558-562.

SUN, X.-F., CARSTENSEN, J.M., ZHANG, H., STÅL, O., WINGREN, S., HATSCHEK, T. & NORDENSKJÖLD, B. (1992). Prognostic significance of cytoplasmic p53 oncoprotein in colorectal adenocarcinoma. *Lancet*, **340**, 1369-1373.

THOR, A.D., MOORE, D.H., EDGERTON, S.M., KAWASAKI, E.S., REIHSHAUS, E., LYNCH, H.T., MARCUS, J.N., SCHWARTZ, L., CHEN, L.-C., MAYALL, B.H. & SMITH, H.S. (1992). Accumulation of p53 tumor suppressor gene protein: an independent marker of prognosis in breast cancers. *J. Natl Cancer Inst.*, **84**, 845-855.

VAN DEN BERG, F.M., TIGGES, A.J., SCHIPPER, M.E.I., DEN HARTOG-JAGER, F.C.A., KROES, W.G.M. & WALBOOMERS, J.M.N. (1989). Expression of the nuclear oncogene p53 in colon tumours. *J. Pathol.*, **157**, 193-199.

VISAKORPI, T., KALLIONIEMI, O.-P., HEIKKINEN, A., KOIVULA, T. & ISOLA, J. (1992). Small subgroup of aggressive, highly proliferative prostatic carcinomas defined by p53 accumulation. *J. Natl Cancer Inst.*, **84**, 883-887.